# Experimental Investigation on the Effects of Photocatalysis in Ultraviolet-Induced Nanoparticle Colloid Jet Machining

**DOI:** 10.3390/ma14051070

**Published:** 2021-02-25

**Authors:** Xiaozong Song, Shundong Ge, Xiaorong Wang, Shengkai Liu

**Affiliations:** School of Mechanical and Electronical Engineering, Lanzhou University of Technology, Lanzhou 730050, China; ggsd137@gmail.com (S.G.); smilewang1997@gmail.com (X.W.); shengkailiu520@gmail.com (S.L.)

**Keywords:** ultraviolet-induced nanoparticle colloid jet machining, photocatalysis, adsorption, polishing efficiency

## Abstract

In this paper, ultraviolet (UV)-induced nanoparticle colloid jet machining is proposed to achieve ultrasmooth surface polishing by using the interaction between nanoparticles and the workpiece surface under the action of the ultraviolet field and the hydrodynamic pressure field. In the process of UV-induced nanoparticle colloid jet machining, the effects of photocatalysis on the interaction between nanoparticles and the workpiece surface need to be further studied in order to better understand the polishing process. This paper presents the interaction between TiO_2_ nanoparticles and a Si workpiece surface with and without ultraviolet irradiation. Scanning electron microscopy (SEM), Fourier-transform infrared spectroscopy (FT-IR), and X-ray photoelectron spectroscopy (XPS) were applied to investigate the differences in the interaction of TiO_2_ nanoparticles with Si workpieces. The SEM and XPS results indicate that the photocatalysis of UV light can promote the interaction between TiO_2_ nanoparticles and a Si surface by creating more interfacial reaction active centers between the TiO_2_ nanoparticles and the Si workpiece. The FT-IR and XPS spectra show that TiO_2_ nanoparticles are chemically bonded to the Si workpiece by oxygen-bridging atoms in Ti-O-Si bonds. Due to the effects of photocatalysis, UV-induced nanoparticle colloid jet machining has a higher polishing efficiency than nanoparticle colloid jet machining with the same polishing parameters.

## 1. Introduction

At present, a variety of surface processing technologies and methods have been developed to meet the urgent needs in optics, electronic science, and other fields related to the creation of ultrasmooth surfaces with high precision, surface figure accuracy, and extremely low surface roughness [1,2]. Nanoparticles have been widely used in various polishing processes to obtain ultrasmooth surfaces. The interaction between nanoparticles and solid surfaces was studied experimentally and theoretically to obtain an atomic smooth surface in the chemical mechanical polishing (CMP) process [3]. The bowl-feed CMP process was reported in Reference [4] to fabricate supersmooth, flat silicon substrates. Cerium-incorporated SBA-15-type nanoparticle abrasives with a larger pore diameter were used on hard disk substrates to achieve a higher material removal rate and lower surface roughness in CMP [5]. In Reference [6], the effects of slurry additives, surfactant, oxidizer, and polyurethane pad on the surface roughness and topography of CMP for silicon were studied, and an atomic-scale ultrasmooth surface was obtained. During photoelectrochemical mechanical polishing, ultraviolet-light irradiation is applied to a wafer surface to accelerate material removal in the polishing process to finish n-type gallium nitride semiconductor wafers [7]. However, CMP is mainly used for the planarization of large planes, which is not suitable for the machining of complex surface workpieces with a small curvature. Nanoparticle colloid jet machining [8] is a polishing technology that can manufacture ultrasmooth surfaces without damage, and it is suitable for polishing complex surface parts with a small curvature. In nanoparticle colloid jet machining, the interaction between the workpiece surface and the nanoparticles is utilized to remove the workpiece surface material at the subnanometer scale [9,10]. However, the extremely low material removal rate limits the wide application of nanoparticle colloid jet machining [11,12]. In order to improve the material removal rate of nanoparticle colloid jet machining and realize the efficient manufacture of ultrasmooth and nondestructive surfaces, a new ultraprecision machining technology called UV-induced nanoparticle colloid jet machining was proposed [13,14,15].

UV-induced nanoparticle colloid jet machining utilizes the interface reaction between the workpiece surface and the nanoparticles under the coupling effect of the ultraviolet field and the jet pressure field to make nanoparticles adsorbing on the workpiece surface [14,15], and the shear viscosity effect of the high-speed colloid jet is used to separate the adsorbed nanoparticles from the workpiece surface together with the atoms of the workpiece surface [16]. When a high-speed TiO_2_ nanoparticle colloid jet is coupled with a UV beam, conduction-band electrons (e^−^) and valence-band holes (h^+^) are created after the excitation of titanium dioxide with UV light [17]. Photogenerated holes (h^+^) and electrons (e^−^) migrate to the surface and react with donor or acceptor species. The photogenerated holes have strong oxidizability and react with H_2_O adsorbed on the surface of the TiO_2_ nanoparticles to create hydroxyl radical (·OH) groups. The scheme can be described by the following basic equations [18]:

TiO_2_ + hv → TiO_2_ (e^−^ + h^+^) (1)

h^+^ + H_2_O_(ads)_ → OH + H^+^ (2)

h^+^ + OH^−^_(ads)_ → OH (3)

Photogenerated electrons (e^−^) can also interact with O_2_ on the surface of the TiO_2_ nanoparticles to create a superoxide radical (O_2_^−^), which is an additional source of ·OH groups. The basic equations of the scheme are as follows [18]:

e^−^ + O_2(ads)_ →O_2_^−^ (4)

O_2_ + H^+^ →HO_2_ (5)

2·O_2_^−^ + 2H_2_O → 2·HO_2_ + 2OH^−^ (6)

2·HO_2_ → O_2_ + H_2_O_2_ (7)

H_2_O_2_ + O_2_^−^ → O_2_ + ·OH + OH^−^ (8)

These ·OH groups demonstrate strong chemical activity and can be easily adsorbed on the surface of the workpiece and the nanoparticles, forming the active center of the interfacial reaction between the nanoparticles and the surface atoms of the workpiece [19]. It is assumed that the atoms of the workpiece surface irradiated by the ultraviolet beam will have stronger chemical activity and react easily with the nanoparticles in the incident colloid. After the interface reaction between the incident nanoparticles and the workpiece surface, new chemical bonds (A-O-B bonds) are generated. As a result, nanoparticles are chemically adsorbed on the workpiece surface. The supposed process can be depicted as follows:

A—OH + OH—B → A—O—B + H_2_O (CH) (9)

where A represents the nanoparticles, and B represents the workpiece surface. H_2_O (CH) is a chemically adsorbed water molecule.

**Figure 1:**
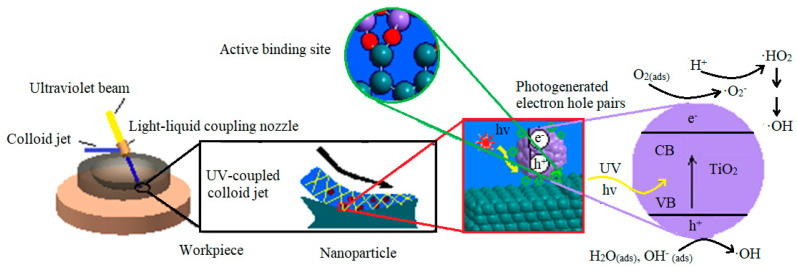
Schematic diagram of UV-induced nanoparticle colloid jet machining.

The shear viscosity of the flowing colloid then causes the chemically adsorbed nanoparticles to separate from the workpiece together with the top atoms of the workpiece surface [16]. In this process, the material of the workpiece surface is removed at an atomic level. Figure 1 shows a schematic diagram of UV-induced nanoparticle colloid jet machining, in which the photochemical effect of UV light is used to fully stimulate and strengthen the interface reaction between the workpiece surface and the nanoparticles in the colloid jet. Therefore, the material removal rate and the manufacturing efficiency of the ultrasmooth surface can be greatly improved.

In this work, the interactions of TiO_2_ nanoparticles with a Si workpiece surface after nanoparticle colloid jet adsorption and UV-induced nanoparticle colloid jet adsorption are reported. The samples of the Si surface adsorbed by the TiO_2_ nanoparticles under different adsorption conditions were fully characterized by SEM, XPS, and FT-IR. The photocatalysis effects of UV light were investigated in the adsorption and polishing experiments.

## 2. Materials and Methods

**Figure 2:**
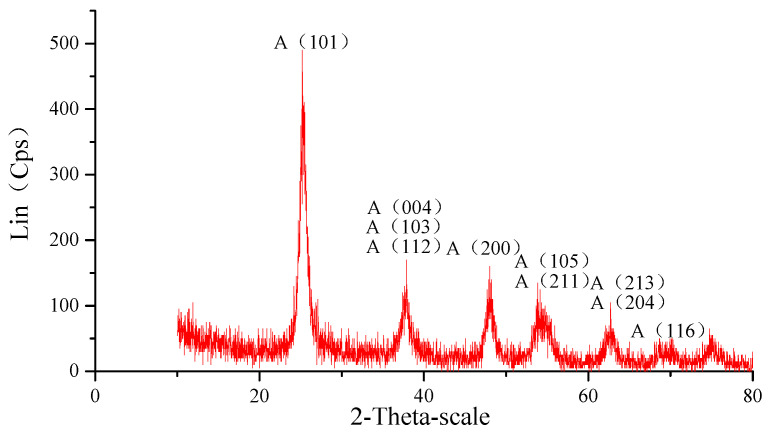
XRD pattern of the TiO_2_ nanoparticles.

**Figure 3:**
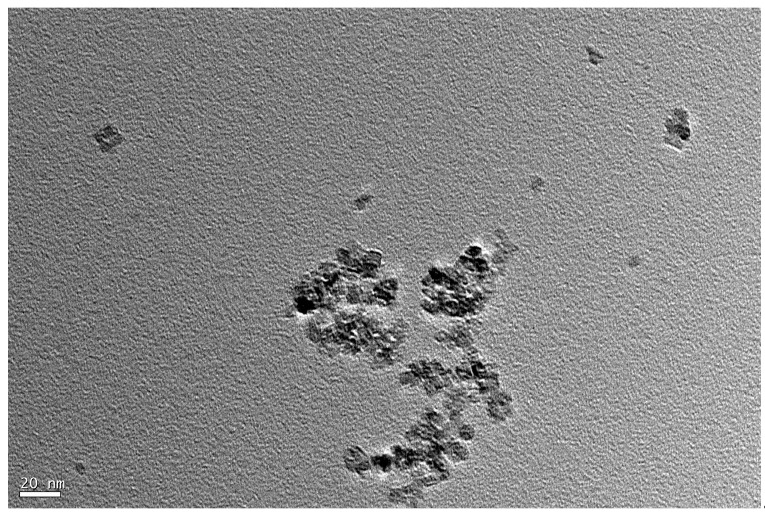
TEM image of the TiO_2_ nanoparticles.

According to the results of the first principles calculation in Reference [19], the main interactions in UV-induced nanoparticle colloid jet machining are as follows: one is the chemical adsorption of the ·OH groups in the colloid on the surface of the nanoparticles and the workpiece; the other is the bonding reaction between the nanoparticles and the hydroxylated workpiece surface. In order to verify the above process, anatase TiO_2_ nanoparticles were used to adsorb on the monocrystalline silicon surface. The TiO_2_ nanoparticles used in the experiment were characterized and detected. Figure 2 shows an X-ray powder diffractometer (XRD) phase analysis result of the TiO_2_ nanoparticles used in the experiments, which was carried out on the D8 ADVANCE XRD (the X-ray source was the Kα ray of a Cu target with a wavelength of 0.15405 nm, the scanning speed was 12°/min, the scanning range was 20–80°, and the sampling interval was 0.02°; Bruker, Karlsruhe, Germany). Morphology studies were carried out using a JEM-1200EX transmission electron microscope (TEM, Jeol Company, Toyoshima, Tokyo, Japan). Figure 3 shows the TEM image of the anatase TiO_2_ nanoparticles. According to the TEM morphology results, the TiO_2_ nanoparticles were not regular spheres but slightly flat irregular flakes, and the size of the TiO_2_ nanoparticles was about 20–30 nm. The TiO_2_ nanoparticles were uniformly dispersed in deionized water to prepare the colloid, and the concentration of the TiO_2_ nanoparticles in the colloid was 10% (volume percentage). Hydrochloric acid and sodium hydroxide were used to adjust the pH value of the colloid, and the adjusted pH value of the colloid was about 7.

**Figure 4:**
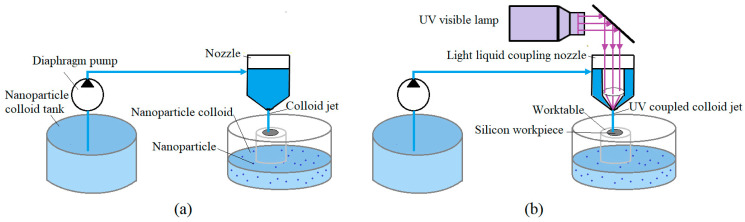
Schematic diagram of the adsorption and polishing experiments. (**a**) Nanoparticle colloid jet adsorption and machining; (**b**) UV-induced nanoparticle colloid jet adsorption and machining.

**Figure 5:**
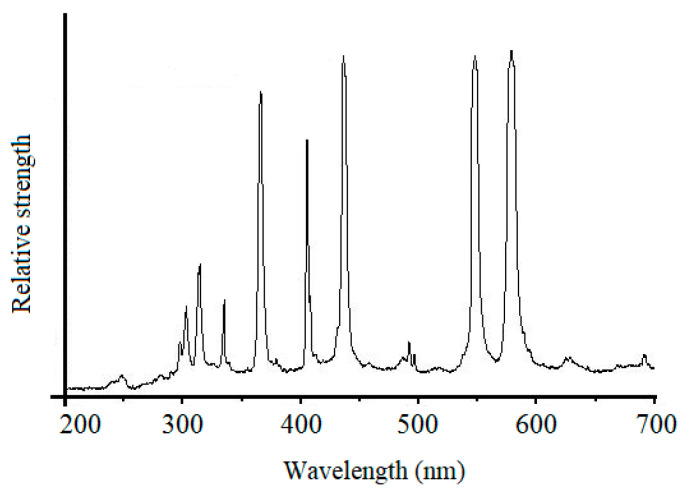
Ultraviolet-visible spectrum of mercury lamp.

In order to verify the reaction mechanism of UV-induced nanoparticle colloid jet machining, comparative experiments were carried out to study the adsorption of the TiO_2_ nanoparticles on the Si workpiece surface under different conditions, as shown in Figure 4. The injection time was 3 min. In the nanoparticle colloid jet adsorption process, the silicon workpiece was firstly fixed on the worktable, and then a diaphragm pump was used to make the nanoparticle colloid enter a nozzle to form a nanoparticle colloid jet beam, which was sprayed on the workpiece surface at a certain speed. In the UV-induced nanoparticle colloid jet adsorption process shown in Figure 4b, the UV light beam was provided by a 500 W high-pressure mercury lamp. The UV light source was turned on to make the UV light pass through a series of optical transmission elements, irradiate on the convex lens entrance of the light-liquid coupling nozzle with a parallel beam, and focus on the nozzle outlet. The UV light beam was then coupled with the nanoparticle colloid jet in the cavity of the light-liquid coupling nozzle to form the UV-coupled colloid jet, and the UV-coupled colloid jet was also sprayed on the workpiece surface. In order to further verify the effects of photocatalysis on the polishing efficiency of UV-induced nanoparticle colloid jet machining, a comparative polishing experiment was carried out. The same Si workpiece was cut into three parts to ensure the same surface roughness, and then 120 min nanoparticle colloid jet machining and UV-induced nanoparticle colloid jet machining experiments were carried out, according to the equipment shown in Figure 4 with the same polishing parameters. In all the above processes, the intensity of pressure was 1 MPa, and the distance between the Si workpiece and the nozzle was about 4 mm. The UV beam was supplied by a 500 W high-intensity mercury lamp. Figure 5 shows the emission spectrum of the high-intensity mercury lamp, and the ultraviolet light at 384 nm was mainly used in this work. The intensity of the light beam at the nozzle outlet was 145 mW/cm^2^. The relevant experimental parameters in this work are listed in Table 1.

## 3. Results 

### 3.1. Characterization of TiO_2_ Nanoparticles Adsorption on the Si Workpiece Surface

**Figure 6:**
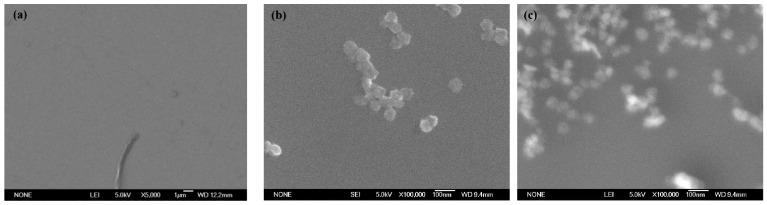
SEM morphology of the TiO_2_ nanoparticles adsorbing on the Si workpieces. (**a**) Original Si surface, (**b**) nanoparticle colloid jet adsorption surface, and (**c**) UV-induced nanoparticle colloid jet adsorption surface.

After 3 min of nanoparticle colloid jet adsorption and UV-induced nanoparticle colloid jet adsorption, the Si workpieces were removed and soaked in deionized water for about 10 s. The Si workpieces were then dried naturally at room temperature. The SEM morphology of the original Si workpiece surface, nanoparticle colloid jet adsorption, and UV-induced nanoparticle colloid jet adsorption Si surface are shown in Figure 6a–c. 

There are no other particles on the Si surface except some machined chips. After three minutes of nanoparticle colloid jet adsorption, a small amount of the TiO_2_ nanoparticles can be observed on the Si workpiece surface. The main reason is that the TiO_2_ nanoparticles adsorbed on the Si surface are taken away by the shear viscosity of the flowing colloid. Under the same conditions, the amount of the TiO_2_ nanoparticles adsorbed on the Si workpiece surface after UV-induced nanoparticle colloid jet adsorption are more than that of nanoparticle colloid jet adsorption.

**Figure 7:**
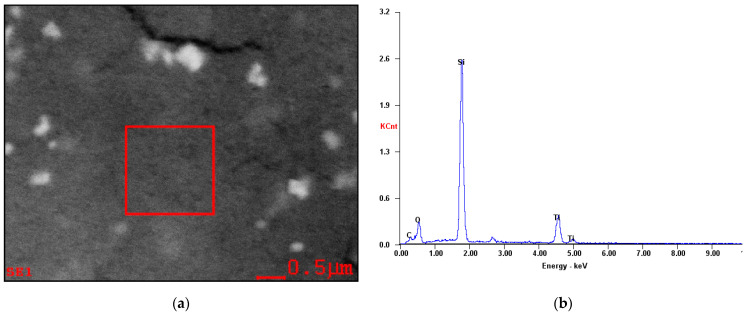
Microanalysis of the Si workpiece surface after UV-induced jet adsorption. (**a**) Areas and (**b**) SEM-EDS microanalysis results.

In order to analyze the element type of the above three adsorption techniques, scanning electron microscope dispersive spectrometer (SEM-EDS) microanalysis was performed. Figure 7a,b show the SEM-EDS microanalysis area and the result of the Si workpiece surface after UV-induced nanoparticle colloid jet adsorption. The SEM-EDS microanalysis result of nanoparticle colloid jet adsorption is the same as the above result. The SEM-EDS microanalysis result reflects that the major element of the microanalysis area is Si, in addition to a small amount of O, Ti, and C elements. 

**Figure 8:**
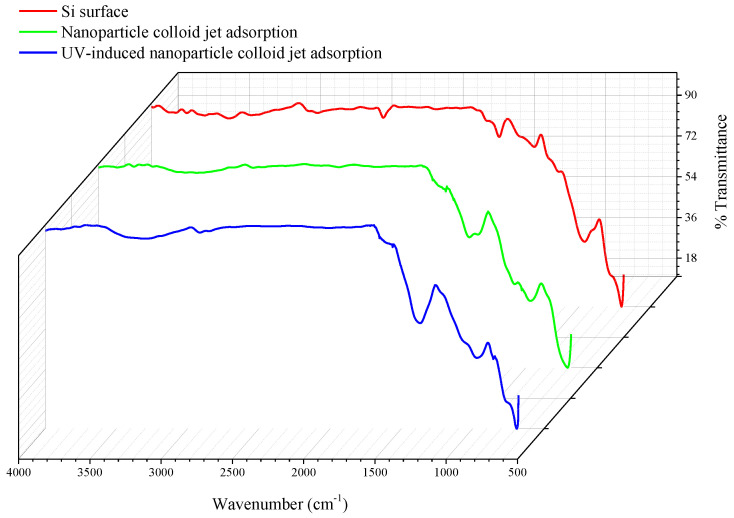
FT-IR of the Si workpiece samples before and after adsorbing on the TiO_2_ nanoparticles.

**Figure 9:**
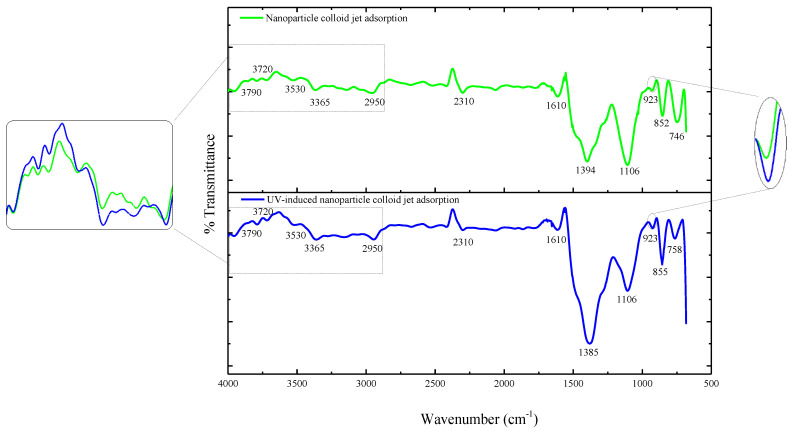
Differential display of the infrared spectrogram of the Si workpiece samples.

The above prepared Si workpiece was characterized by Fourier-transform infrared spectroscopy (FT-IR, Nexus 670 FT-IR spectrometer, Nicoli, Madison, WI, USA). Figure 8 displays the FT-IR spectra of the three Si workpiece samples. In order to accurately characterize the differences in the infrared spectra of the Si workpiece surface before and after the adsorption of the TiO_2_ nanoparticles, the FT-IR differential spectra were obtained to deduct the original bands of the Si workpiece surface and show the new bands after the adsorption of the TiO_2_ nanoparticles. The FT-IR differential spectra of nanoparticle colloid jet adsorption and UV-induced nanoparticle colloid jet adsorption are shown in Figure 9. 

The bands at 746–758 cm^−1^ in the above two samples can be attributed to the Ti-O band, indicating that there are TiO_2_ nanoparticles adsorbed on both Si workpiece samples. The bands at 852–855, 1106, and 1385–1394 cm^−1^ are attributed to the stretching vibration of the Si-O band. The bands at 3790, 3720, and 3365–2950 cm^−1^ are attributed to the stretching vibration of the OH band. The band at 1610 cm^−1^ belongs to the bending vibration of the ·OH group. The partially enlarged comparison shows that there are no significant changes in the position of the ·OH group bands in nanoparticle colloid jet adsorption and UV-induced nanoparticle colloid jet adsorption, but the absorption intensity of the ·OH group increases obviously after UV irradiation. The band at 2320 cm^−1^ corresponds to the stretching vibration of Si-H, which indicates that the adsorption of H occurs simultaneously with the adsorption of the ·OH group in the colloid environment. In the frequency region of the M-O-M group (900–1500 cm^−1^), the new band at 923 cm^−1^ in both samples can be attributed to the Ti-O-Si stretching vibration band [20]. The relative intensity of the Ti-O-Si band in UV-induced nanoparticle colloid jet adsorption is stronger than that of nanoparticle colloid jet adsorption.

**Figure 10:**
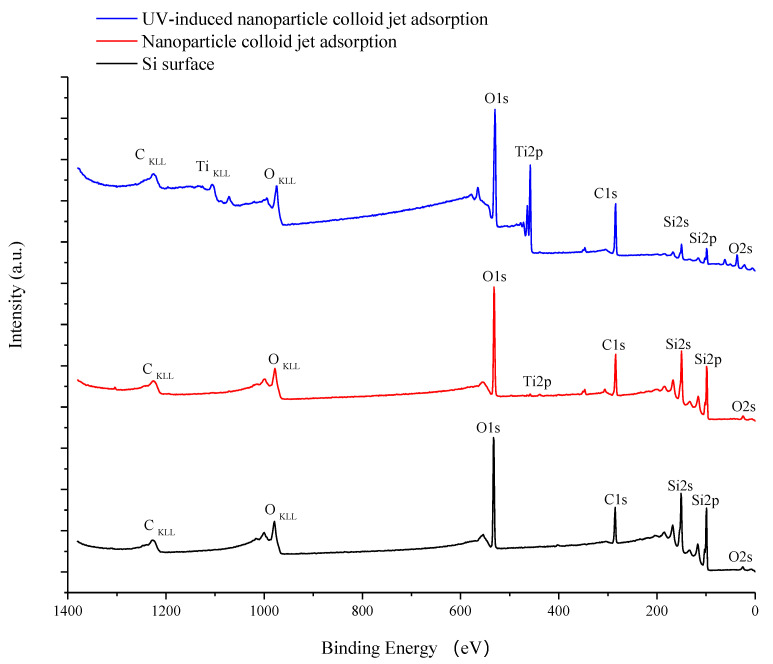
Full XPS spectrum of the three Si workpiece surface samples.

X-ray photoelectron spectroscopy (XPS, ESCALAB 250Xi, ThermoFisher Scientific/monochromatic Al target, Massachusetts, USA) was also performed to investigate the corresponding coordination states on the three Si workpiece samples. According to the full XPS spectrum of the three samples shown in Figure 10, the elements on the original Si workpiece surface are Si, C, and O. After 3 min of nanoparticle colloid jet adsorption and UV-induced nanoparticle colloid jet adsorption, Si, O, Ti, and C elements could be detected on both workpiece samples, which is consistent with the results of SEM-EDS microanalysis. According to the relative content of major elements on the test samples shown in Table 2, the relative content of Ti on the nanoparticle colloid jet adsorption surface is about 0.39%, and that on the UV-induced nanoparticle colloid jet adsorption surface is 12.36%.

**Figure 11:**
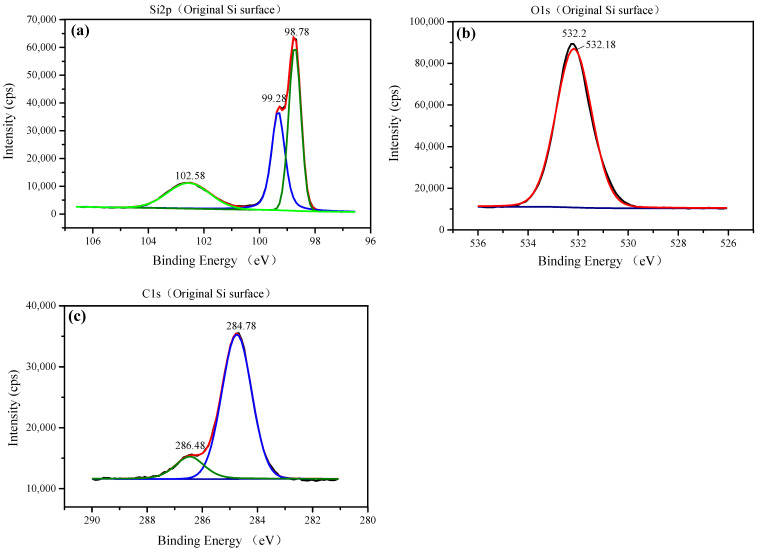
XPS spectra of the original Si surface: (**a**) Si2p, (**b**) O1s, and (**c**) C1s.

**Figure 12:**
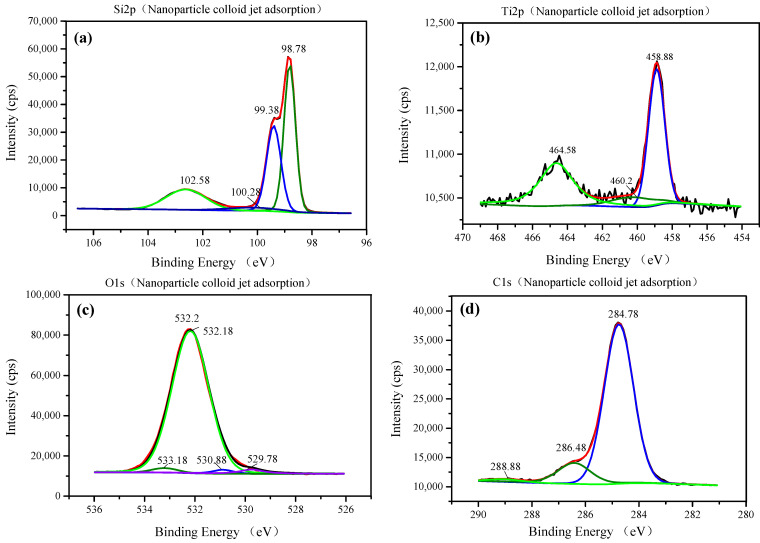
XPS spectra of nanoparticle colloid jet adsorption Si surface: (**a**) Si 2p, (**b**) Ti 2p, (**c**) O 1s, and (**d**) C 1s.

**Figure 13:**
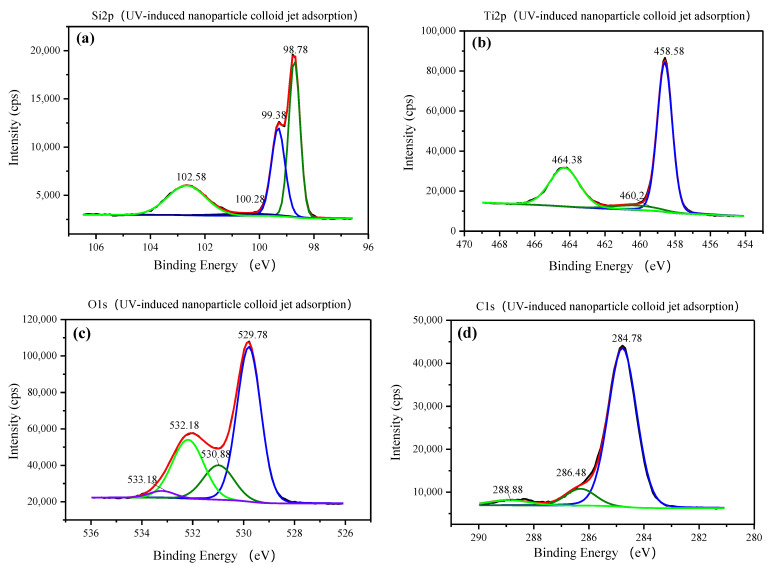
XPS spectra of the UV-induced nanoparticle colloid jet adsorption Si surface: (**a**) Si 2p, (**b**) Ti 2p, (**c**) O 1s, and (**d**) C 1s.

Using the binding energy of the contaminated carbon electron (284.78 eV) as the internal standard, the binding energies of the elements in the three samples were obtained, as shown in Figure 11, Figure 12 and Figure 13, respectively. For the original Si surface sample, the high-resolution photoelectron spectrum of the Si 2p shown in Figure 11a is fitted by three peaks falling at 98.78, 99.28, and 102.58 eV. According to the standard XPS spectrum, the binding energies falling at 98.78 and 99.28 eV belong to Si, and the binding energy falling at 102.58 eV belongs to SiO_2_ [21,22,23]. From the strength of the two peaks, it can be seen that most of the Si elements on the original silicon surface exist in the form of silicon and a small part in the form of silicon dioxide. Figure 11b shows the O 1s core line fitted by only one peak of 532.18 eV [24,25,26], which is the spectral position of the oxygen component corresponding to the Si 2p component associated with SiO_2_ falling at 102.58 eV on the Si workpiece surface. As shown in Figure 11c, the component of C falls at 284.78 and 286.48 eV. The C element on the original Si workpiece surface may be from environmental C pollution and the residual in the preprocess of the Si workpiece.

As shown in Figure 12a and Figure 13a, for the nanoparticle colloid jet adsorption and UV-induced nanoparticle colloid jet adsorption samples, the high-resolution photoelectron spectrum of the Si 2p is fitted by four peaks falling at 98.78, 99.38, 100.28, and 102.58 eV. There are three peaks that are the same as those on the original Si surface, but the peak falling at 100.28 eV is a new peak generated in the adsorption process of TiO_2_ nanoparticles on the Si workpiece surface. Figure 12b and Figure 13b show the high-resolution photoelectron spectrum of the titanium on the nanoparticle colloid jet adsorption and UV-induced nanoparticle colloid jet adsorption samples. The Ti 2p core line is composed of a doublet that describes the typical spin–orbit splitting of the 2p level in the 2p1/2 and 2p3/2 components. The Ti 2p core line is fitted by three peaks falling at 458.58–458.88, 460.2, and 464.38–464.58 eV. The two spin–orbit peaks at 458.58–458.88 and 464.38–464.58 eV correspond to Ti in the anatase TiO_2_ form [20,27,28], and the peak at 460.2 eV is also a new peak generated in the interaction between the TiO_2_ nanoparticles and the Si workpiece surface. According to the O 1s core lines shown in Figure 12c and Figure 13c, the O 1s core line is fitted by four peaks falling at 529.78, 530.88, 532.18, and 533.18 eV. The peak at 532.18 eV is inherited from the original Si surface, and the other three peaks are newly introduced in the adsorption process. The peak at 529.78 eV is the spectral position of the oxygen component corresponding to the Ti 2p component associated with TiO_2_ falling at 458.88 eV. The peak at 530.88 eV is the spectral position of the oxygen component corresponding to the Ti peak at 460.2 eV and the Si peak at 100.28 eV. The last component at 533.18 eV is the spectral position of the oxygen component corresponding to the C 1s component falling at 288.88 eV shown in Figure 12d and Figure 13d, which may be from the dispersant and surfactant in the nanoparticle colloid solution. Table 3 shows the position of the Si 2p, Ti 2p, C 1s, and the correspondent O 1s.

### 3.2. Polishing Efficiency Comparative Study 

**Figure 14:**
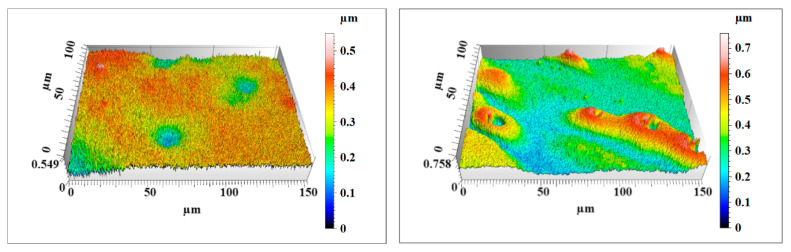
The original Si workpiece surface morphology.

**Figure 15:**
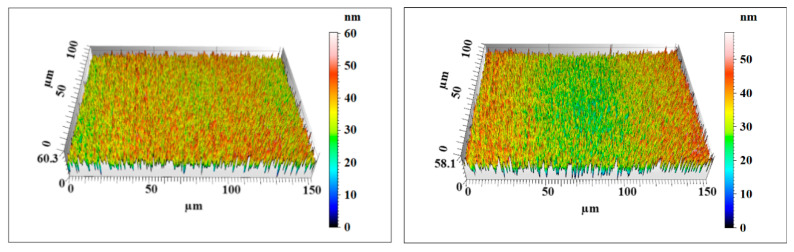
The nanoparticle colloid jet machining Si workpiece surface morphology.

**Figure 16:**
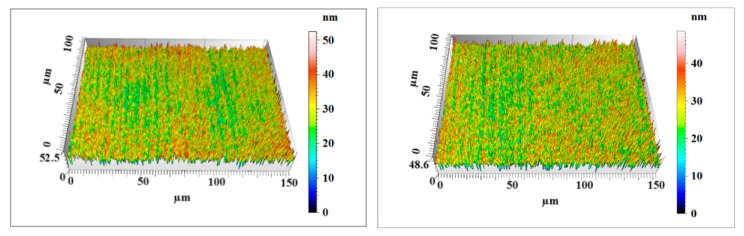
The UV-induced nanoparticle colloid jet machining Si workpiece surface morphology.

After 120 min of comparative polishing experiments, the two Si workpieces were rinsed with a water jet to completely remove the remaining nanoparticles on the surface. The surface morphology and roughness of the Si workpiece were characterized by a noncontact three-dimensional surface profilometer (MicroXAM-800, KLA-Tencor, Milpitas, CA, USA). Two measuring points were randomly selected on the surface of each workpiece with a measurement area of 150 μm × 115 μm. Figure 14, Figure 15 and Figure 16 show the surface morphology of the three Si workpieces before and after polishing. The surface roughness values of random measuring points on each workpiece surface are listed in Table 4. The experiment results only reflect the relative material removal efficiency of each polishing method through the decrease of surface roughness under the same polishing conditions but do not reflect the lowest surface roughness that these polishing methods can achieve.

According to the measuring results, a large number of microsurface peaks and pits can be observed on the original Si surface. The average maximum p-v value (Sz) is about 653 nm, and the average surface roughness is Sq 84.3 nm (Sa 66.8 nm). After polishing by nanoparticle colloid jet machining, a small number of microsurface peaks and pits remain on the Si workpiece. The average maximum p-v value (Sz) of the Si workpiece polished by nanoparticle colloid jet machining is 59.2 nm, and the average surface roughness is decreased to Sq 7.575 nm (Sa 6.025 nm). The surface morphology results of the Si workpiece after polishing by UV-induced nanoparticle colloid jet machining show that most of the microsurface peaks and pits were removed away from the workpiece. The average maximum p-v value (Sz) is decreased to 50.5 nm, and the average surface roughness is reduced to Sq 5.58 nm (Sa 4.44 nm). Without considering the influence of other factors, the material removal efficiency of the two polishing methods can be simply expressed by the relative changes of surface roughness values. Under the same conditions of 120 min polishing, the surface roughness of the UV-induced nanoparticle colloid jet machining is lower, and the workpiece surface is flatter and more uniform.

## 4. Discussion

According to the SEM morphology results, there are more TiO_2_ nanoparticles adsorbed on the Si workpiece surface of UV-induced nanoparticle colloid jet adsorption. The XPS relative content of the Ti element on the Si workpiece surface also demonstrates that the amount of TiO_2_ nanoparticles adsorbed on the silicon surface in UV-induced nanoparticle colloid jet adsorption is much larger than that of nanoparticle colloid jet adsorption. These results prove that the photocatalysis of UV light can excite and strengthen the interaction between TiO_2_ nanoparticles and the Si surface in colloid. The FT-IR spectra results show that the ·OH groups in the colloid interact with the atoms of the Si workpiece surfaces and TiO_2_ nanoparticles to achieve surface adsorption and generate surface ·OH groups in both processes of nanoparticle colloid jet adsorption and UV-induced nanoparticle colloid jet adsorption. The position of the ·OH group bands is unchanged with and without UV irradiation, but the absorption intensity of the OH group increases after UV irradiation, which indicates that the concentration of the ·OH group is greatly enhanced. The new band at 923 cm^−1^ is attributed to the Ti-O-Si stretching vibration band, and the relative intensity of the Ti-O-Si band in UV-induced nanoparticle colloid jet adsorption is stronger than that of nanoparticle colloid jet adsorption. The test results can be regarded as evidence of the interaction between the TiO_2_ nanoparticles and the Si workpiece surface in nanoparticle colloid jet machining and can further prove the promotion effect of photocatalysis in the interaction between TiO_2_ nanoparticles and the Si surface in the colloid. The XPS fitting peaks falling at 100.28, 460.2, and 530.88 eV indicate that there is chemical bonding between the TiO_2_ nanoparticles and the Si workpiece surface in both nanoparticle colloid jet machining and UV-induced nanoparticle colloid jet machining, and the TiO_2_ nanoparticles are bonded to the Si workpiece surface through a Ti-O-Si bond. These results are consistent with the results of the FT-IR spectra analysis. The comparative polishing experiment results show that due to the effects of photocatalysis, the polishing efficiency of UV-induced nanoparticle colloid jet machining is higher than that of nanoparticle colloid jet machining under the same polishing conditions. 

## 5. Conclusions

The photocatalysis of UV light can create ·OH groups in TiO_2_ nanoparticles colloid. These ·OH groups in the colloid can easily adsorb on the surface of the workpiece and the nanoparticles, increasing the number of surface OH groups. The surface ·OH groups are the active center of the interfacial reaction between the nanoparticles and the workpiece, which can promote the adsorption of TiO_2_ nanoparticles on the Si workpiece surface. In the interface reaction process, the TiO_2_ nanoparticles are chemically bonded to the Si workpiece surface by generating Ti-O-Si bonds. Due to the effects of photocatalysis, there are more chemical reaction active centers on the surface of the nanoparticles and the workpiece in the UV-induced nanoparticle colloid jet machining, and the probability of an interfacial reaction between the nanoparticles and workpiece surfaces is greater. As a result, UV-induced nanoparticle colloid jet machining has a higher polishing efficiency than nanoparticle colloid jet machining with the same polishing parameters. 

## Figures and Tables

**Table 1:** Relevant parameters in the adsorption and polishing experiments.

Experimental Conditions	Value
Workpiece material	Si
Nanoparticles material	TiO_2_
Diameter of nanoparticles	20–30 nm
pH of colloid	7
Concentration of colloid	10% (volume percentage)
Intensity of pressure	1 MPa
Light intensity	145 mW/cm^2^
Injection distance	4 mm
Nozzle diameter	1 mm
Injection time	3 min
Polishing time	120 min

**Table 2:** Relative content of some major elements on each test sample.

Element (Atomic %)	Original Si Surface	Nanoparticle Colloid Jet Adsorption Surface	UV-Induced Nanoparticle Colloid Jet Adsorption Surface
Si	46.14	41.74	13.35
Ti	-	0.39	12.36
O	30.47	31.79	41.09
C	23.39	26.08	33.2

**Table 3:** Position of the Si 2p, Ti 2p, C 1s, and the correspondent O1s (all values are in eV).

Sample	Si 2p	Ti 2p	C 1s	O 1s
Original Si surface	98.78	-	284.78	532.18
	99.28			
	102.58		286.48	
Nanoparticle colloid jet adsorption surface and UV- induced nanoparticle colloid jet adsorption surface	98.78	458.58–458.88	284.78	529.78
	99.28–99.38	460.2	286.48	530.88
	100.28	464.38–464.58	288.88	532.18
	102.58			533.18

**Table 4:** Surface roughness of the three Si workpieces (all values are in nm).

Workpiece	Surface Roughness	Measuring Point 1	Measuring Point 2	Average Value
Original Si workpiece	Sz	549	758	653.5
	Sq	65.6	103	84.3
	Sa	50.1	83.5	66.8
Nanoparticle colloid jet machining workpiece	Sz	58.1	60.3	59.2
	Sq	7.78	7.37	7.575
	Sa	6.20	5.85	6.025
UV-induced nanoparticle colloid jet machining workpiece	Sz	52.4	48.6	50.5
	Sq	5.74	5.42	5.58
	Sa	4.57	4.31	4.44

## Data Availability

The data presented in the article are available from the corresponding author.
